# Using a Network-Based Analysis Approach to Investigate the Involvement of *S. aureus* in the Pathogenesis of Granulomatosis with Polyangiitis

**DOI:** 10.3390/ijms24031822

**Published:** 2023-01-17

**Authors:** Gregory Rowland, Andreas Kronbichler, Rona Smith, David Jayne, Piet H. van der Graaf, Vijayalakshmi Chelliah

**Affiliations:** 1 Certara QSP, Certara UK Limited, Sheffield, S1 2BJ, UK; 2 Department of Medicine, University of Cambridge, Cambridge CB2 0QQ, UK; 3 Leiden Academic Centre for Drug Research, University of Leiden, 2311 EZ Leiden, The Netherlands

**Keywords:** granulomatosis with polyangiitis, ANCA associated vasculitis, *Staphylococcus aureus*, network-based analysis, disease network, molecular signatures, biological processes

## Abstract

Chronic nasal carriage of *Staphylococcus aureus* (SA) has been shown to be significantly higher in GPA patients when compared to healthy subjects, as well as being associated with increased endonasal activity and disease relapse. The aim of this study was to investigate SA involvement in GPA by applying a network-based analysis (NBA) approach to publicly available nasal transcriptomic data. Using these data, our NBA pipeline generated a proteinase 3 (PR3) positive ANCA associated vasculitis (AAV) disease network integrating differentially expressed genes, dysregulated transcription factors (TFs), disease-specific genes derived from GWAS studies, drug–target and protein–protein interactions. The PR3+ AAV disease network captured genes previously reported to be dysregulated in AAV associated. A subnetwork focussing on interactions between SA virulence factors and enriched biological processes revealed potential mechanisms for SA’s involvement in PR3+ AAV. Immunosuppressant treatment reduced differential expression and absolute TF activities in this subnetwork for patients with inactive nasal disease but not active nasal disease symptoms at the time of sampling. The disease network generated identified the key molecular signatures and highlighted the associated biological processes in PR3+ AAV and revealed potential mechanisms for SA to affect these processes.

## 1. Introduction

Granulomatosis with polyangiitis (GPA) is a rare systemic autoimmune disease characterised by vasculitis of small and medium-sized vessels and necrotising granulomatous lesions. GPA belongs to a group of diseases termed anti-neutrophil cytoplasmic antibody (ANCA)-associated vasculitis (AAV) which also includes eosinophilic granulomatosis with polyangiitis (EGPA) and microscopic polyangiitis (MPA). Central to AAV is the presence of autoantibodies against two proteins—proteinase-3 (PR3) and myeloperoxidase (MPO) predominately expressed in neutrophils [1].

Recent findings have suggested that serological classification (PR3 vs. MPO) of AAV patients may be more robust than traditional clinical phenotyping (GPA vs. MPA) [2]. GPA is more frequently associated with PR3-ANCA, and commonly involves the upper and lower respiratory tract as well as the kidneys. Nasal involvement is seen in up to 80% of GPA patients [3]. A mixture of environmental and genetic factors are implicated in the aetiology of GPA [1]. An environmental factor of particular interest is nasal colonisation of *Staphylococcus aureus* (SA). Chronic nasal carriage of SA has been shown to be significantly higher in GPA patients when compared to chronic rhinosinusitis with nasal polyps and healthy subjects, as well as being associated with increased endonasal activity and disease relapse [4,5,6]. These data, alongside evidence that treatment with the antibiotic trimethoprim/sulfamethoxazole can reduce disease relapses in patients with localised GPA, point towards a pathogenic role for SA in GPA [7,8].

In recent years, network-based analysis (NBA) has gained significant interest in drug discovery and development for analysing and predicting novel targets and disease mechanisms using multifaceted biological data [9]. To date, an exact mechanism detailing SA involvement in GPA has not been found [5]. In this study, we use an NBA approach combining prior knowledge of human–human and human–pathogen protein–protein interactions (PPIs), gene regulatory networks, genome-wide association studies (GWAS) and drug–target interactions with publicly available transcriptomic data to identify dysregulated genes and biological processes (functional modules) in PR3+ AAV. Moreover, we highlight potential mechanisms through which SA may impact GPA via its interaction with these modules and how both nasal disease activity and immunosuppressant treatment affect their expression.

## 2. Results

### 2.1. Generating the PR3+ AAV Disease Network

**Figure 1:**
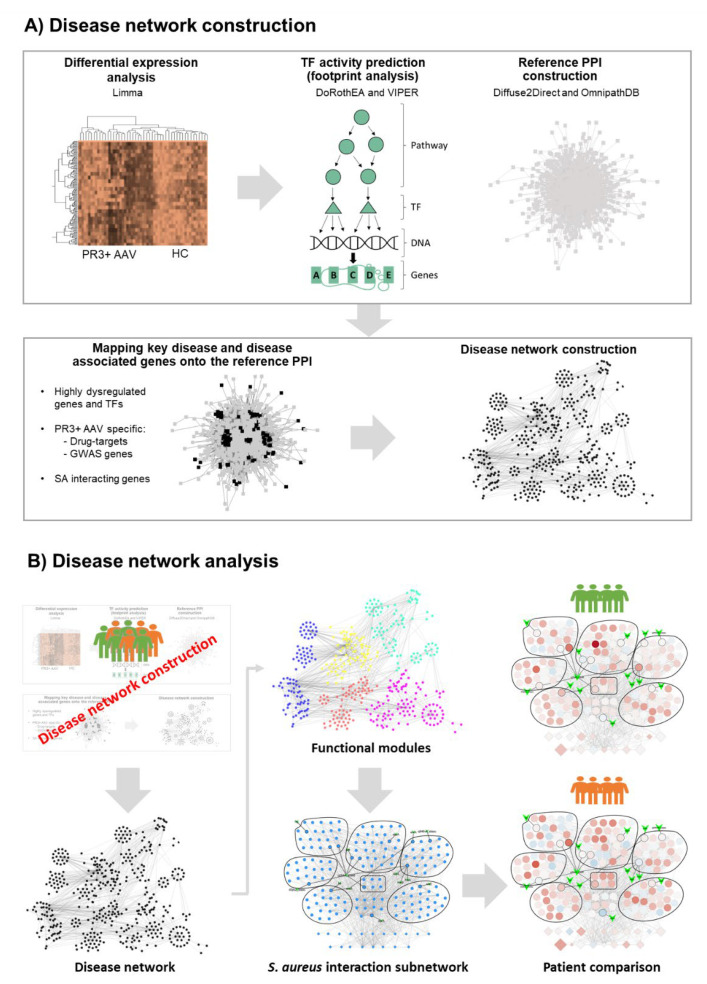
Applying network-based analysis (NBA) to investigate the role of S. aureus in granulomatosis with polyangiitis (GPA). (**A**) Disease network construction. Our NBA pipeline used publicly available nasal transcriptomics to carry out differential gene expression analysis comparing PR3+ AAV patients versus healthy controls (HC). This was performed using mixed linear regression models via the limma R package. The activity of transcription factor (TFs) was estimated by the VIPER R package using limma moderated t-scores and TF regulatory networks provided by the DoRothEA database. A reference network of directed protein–protein interactions (PPIs) was assembled using PPIs listed in the OmnipathDB and Diffuse2Direct databases. Disease genes and disease associated genes including dysregulated genes and TFs, genes encoding drug-targets of drugs used clinically or investigationally to treat GPA, genes associated with PR3+ AAV, GPA or AAV in genome wide association studies (GWAS) and genes encoding proteins reported to interact with *S. aureus* (SA) toxins in the Host Pathogen Interaction Database were mapped onto their corresponding proteins in the reference PPI network. The selected disease genes and their interactions were used to construct the PR3+ AAV disease network. (**B**) Disease network analysis. Functional enrichment analysis was used to find significantly enriched biological processes and pathways (functional modules) in the PR3+ AAV network. The functional modules in the PR3+ AAV disease network which contained the highest number of SA interacting genes were used to create an *S. aureus* interaction subnetwork. The expression levels of the genes in this subnetwork were compared across different patient subgroups.

To create a network of genes whose dysregulation are implicated in PR3+ AAV (i.e., a disease network), we integrated data from a range of sources including human–human and human–pathogen PPIs, GWAS, drug–target interactions, gene regulatory networks and transcriptomics (described in full in the Materials and Methods and summarised in Figure 1). Transcriptomics (in the form of microarray intensities) were the primary guiding source in deciding which genes were incorporated into the disease network. Whole-genome gene expression data derived from nasal brushings were accessed via the Gene Expression Omnibus series GSE119136 [10]. This study was chosen due to our interest into how nasal colonisation by SA may play a role in GPA pathogenesis and relapse—thus transcriptomics derived from the site of colonisation were considered most relevant. A subset of samples from this study was selected including 26 PR3+ AAV patients—all of whom were PR3-ANCA positive during their disease course—and 12 healthy controls. At the time of sampling, 8/26 PR3+ AAV patients had active nasal disease, 11/26 had no nasal activity but had a history of nasal involvement (inactive) and 7/26 had no current or historical nasal involvement (non-nasal). Table 1 summarises the baseline characteristics and treatment of patients with PR3+ AAV.

### 2.2. PR3+ AAV Disease Network Captures Key Disease Genes

A PR3+ AAV disease network comprising 758 genes and 6008 interactions was generated. Captured in this network were a number of important genes, which have previously been reported to be dysregulated in GPA (summarised by Kronbichler et al. in [11]). For example, genes involved in priming and activation of key immune effector cells, e.g., IL1B, IL2RA, IL10, IL32, TNF, MMP9, CD14 and SERPINA1—all of which were seen to be upregulated when compared to healthy controls (Supplementary Table S1). Moreover, genes associated with extracellular matrix remodelling such as TIMP1 and MMP9 as well as endothelial injury and leukocyte adhesion such as S100A8/A9, ITGAM, ITGB2, ITGAX and ICAM1 were all upregulated in our analysis reflecting the results of prior studies.

### 2.3. Clustering Reveals Differences between Patient Subgroups

**Figure 2:**
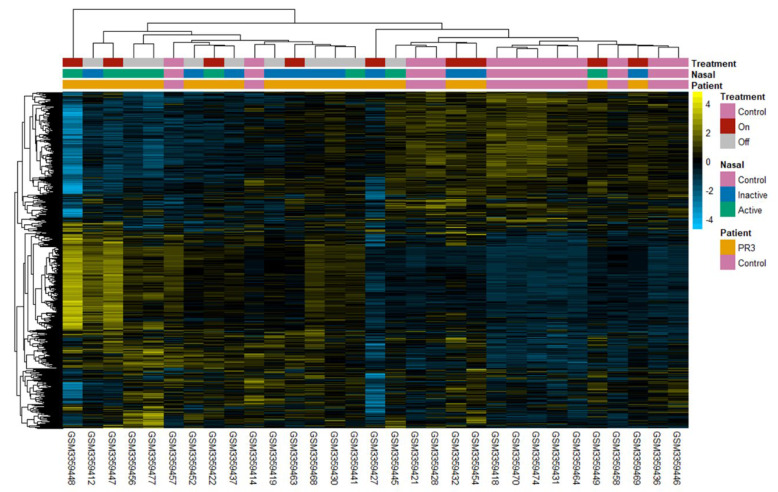
Expression heatmap of disease network genes. Disease network genes (rows) and PR3+ patients (columns) were hierarchically clustered using the Euclidean distance. Gene expression was scaled across samples with yellow/blue representing high/low expression (as shown in the key). Patient groupings were annotated using three colour bars. Healthy controls were shown in pink for all classifications. The top colour bar shows patients on/off treatment in red/grey. The middle colour bar shows patients nasal disease activity with active nasal disease in green and inactive in blue. The bottom colour bar shows disease status with GPA patients shown in orange.

Unsupervised clustering of samples based on the expression of genes in the PR3+ AAV disease network genes revealed differences between healthy controls and PR3+ AAV patients as well as within PR3+ AAV patient subgroups (Figure 2). Three levels of comparisons were made (represented in the colour bars of Figure 2). Firstly, disease state, i.e., PR3+ AAV or healthy control; secondly, nasal disease activity at the time of sampling (active or inactive) and lastly on the treatment status of the patients with respect to immunosuppressant treatment (excluding prednisolone). Clustering was unable to perfectly separate healthy controls and disease patients, however, all PR3+ AAV patients clustering closest to healthy controls except one were receiving treatment with a majority having inactive nasal disease.

### 2.4. SA-PR3+ AAV Interaction Network Highlights Potential Impact of SA on Key Functional Modules

**Figure 3:**
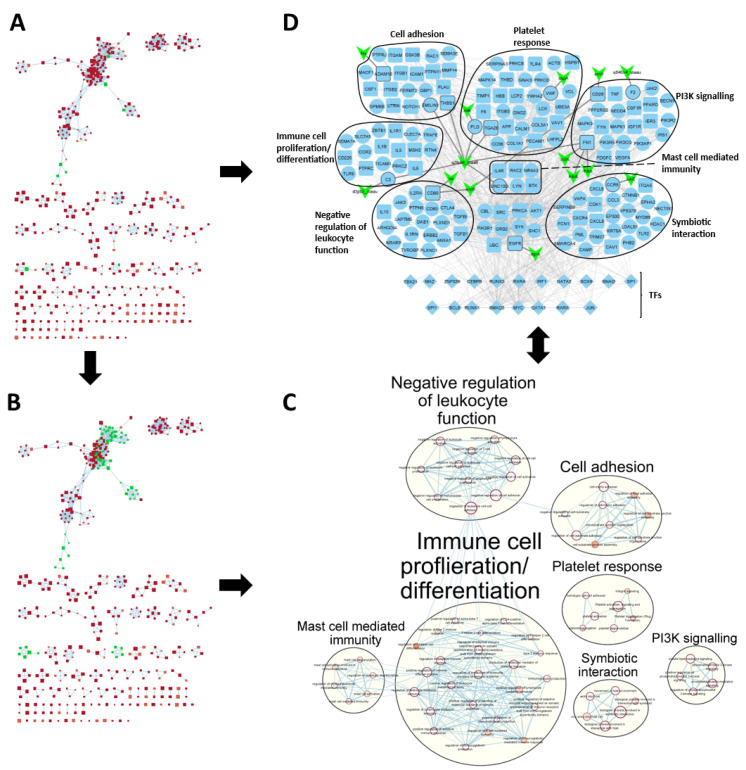
Generating the SA-PR3+ AAV interaction network from the disease network. (**A**) Network of significantly enriched biological processes/pathways (red nodes) in disease network genes. Edges represent shared genes between terms. Highlighted in green are the terms with the highest number of SA interacting genes. Disease network genes associated with the highlighted terms made up a subnetwork shown in (**D**). (**B**) To find terms related to those affected by interactions with SA, the direct neighbours of the highlighted nodes were selected and subsequently coloured green. The highlighted terms in (**B**) were clustered and labelled in (**C**).

To investigate the role of SA in GPA, we focussed on the functional modules within our PR3+ AAV network, which may also be affected by interactions with SA (Figure 3). This was done by first finding significantly enriched Gene Ontology Biological Process (GO: BP) and Reactome pathway terms in the PR3+ AAV disease network (Figure 3A). Next, terms containing the highest number of SA interacting genes (SA terms) were selected and their associated genes in the disease network were used to create the SA-PR3+ AAV interaction network (Figure 3D). Lastly, SA terms and terms closely related to them were clustered and annotated highlighting the main biological themes in the SA-PR3+ AAV interaction network (Figure 3B,C).

In total, 172 genes and 930 interactions from the PR3+ AAV disease network were used to create the SA-PR3+ AAV interaction network (Figure 3D). In addition, 21 SA-human interactions derived from the Host-Pathogen Interaction Database (HPIDB) involving 13 SA virulence factors were included [12]. Unsurprisingly, many of the genes in the network were associated with host-pathogen/-symbiont interactions and immune responses, e.g., immune cell proliferation/differentiation, negative regulation of leukocyte function and mast cell mediated immunity. Alongside these, genes associated with cell-matrix/-cell adhesion, PI3K signalling and the platelet response were present in the network. Moreover, a range of SA virulence factors were represented in the network including exoenzymes, exotoxins and SA surface proteins. 

### 2.5. Treatment Induced Expression Pattern Is Dependent on Nasal Disease Status

**Figure 4:**
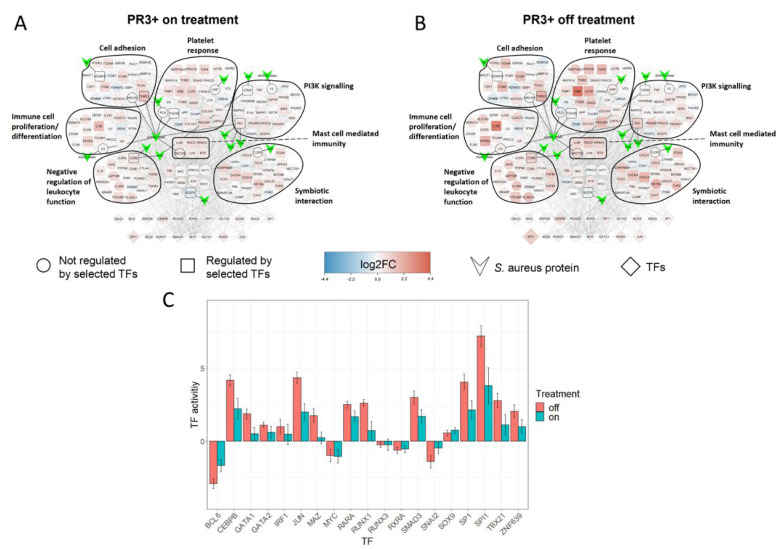
Comparison of SA-PR3+ AAV interaction networks between on and off treatment groups (excluding prednisone). (**A**,**B**) SA-PR3+ AAV interaction networks with nodes representing different classes of genes (as specified in the key) and edges representing protein–protein interactions (PPIs). The size of each TF was mapped to their estimated activity. Reduced differential expression and TF activity observed in those treated by immunosuppressants. (**C**) Bar chart showing average TF activity in on/off treatment groups. The bars were coloured green/red representing patients on/off treatment. Error bars show the standard error of the mean.

**Figure 5:**
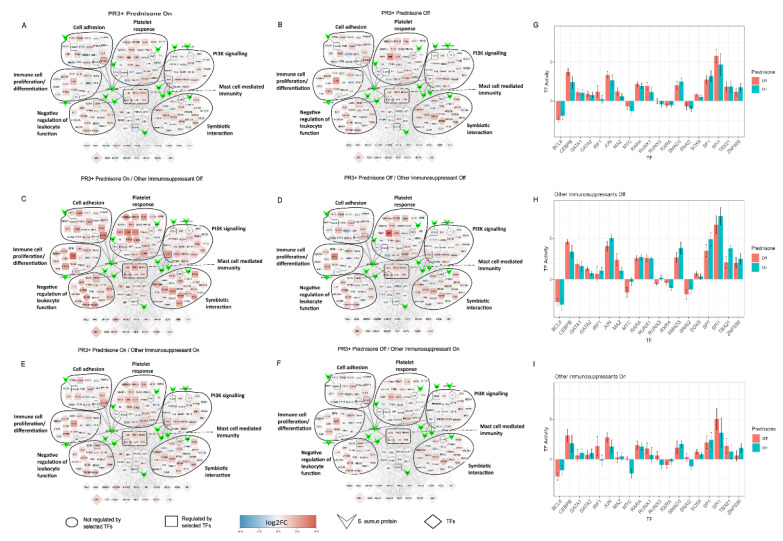
Comparison of SA-PR3+ AAV interaction networks between prednisone on and off treatment groups. (**A**–**F**) Networks and bar charts are as defined in Figure 4. (**A**,**B**) Patients with prednisone showed a slightly increased differential expression when compared to off treatment, suggesting genes in the network remain dysregulated despite being treated with prednisone. (**C**,**D**) Patients with or without prednisone and without the concomitant use of other immunosuppressants. Patients receiving prednisone alone (no other immunosuppressants) have highly dysregulated genes when compared to treatment-naïve patients. A similar trend as in A and B is seen. (**E**,**F**) Patients with or without prednisone but with other immunosuppressants. Other immunosuppressant treatment either mono or in combination with prednisone showed decreased differential expression levels, i.e., decreased dysregulation suggesting that other immunosuppressant treatment on its own or in combination with prednisone might revert the dysregulation. (**G**–**I**) TF activities for prednisone on/off (**G**) with immunosuppressants on/off (**H**–**I**) reflects the changes observed in the SA-PR3+ AAV interaction networks for the respective patient groups.

**Figure 6:**
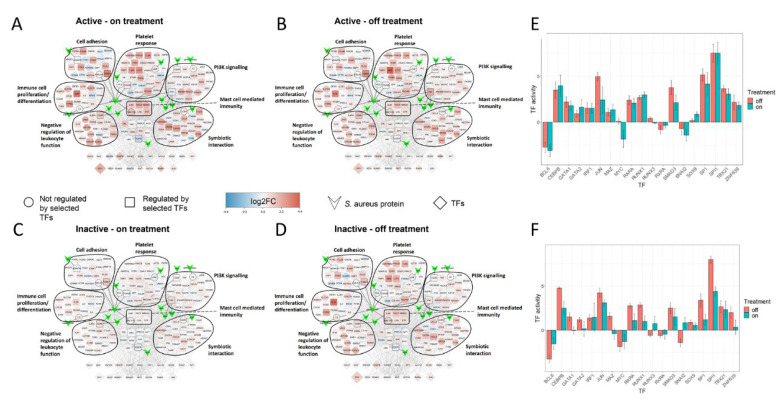
Comparison of SA-PR3+ AAV interaction network across different nasal disease and treatment groups. (**A**–**F**) Networks and bar charts are as defined in Figure 4. (**A**,**B**) Active nasal disease patients on and off treatment show little difference in expression suggesting genes in the network remain dysregulated despite being on treatment. (**C**,**D**) Inactive nasal disease patients show a reduction in differential expression for those on treatment. (**E**,**F**) TF activities for active/inactive and on/off treatment reflect the changes observed in the SA-PR3+ AAV interaction networks for the respective patient groups.

Average differential expression levels versus healthy controls in the SA-PR3+ AAV interaction network showed reduced levels of differential expression in patients receiving immunosuppressant (n = 16) treatment when compared to those off immunosuppressants (excluding prednisolone) (n = 10) (Figure 4). We then compared the differential expression levels between current prednisone-users (n = 13) and non-users (n = 13). Patients receiving prednisone showed slightly increased differential expression levels when compared to those with steroids, suggesting that genes in the network are being dysregulated despite the treatment with prednisone (Figure 5A,B). We then further classified the patients based on prednisone (on/off) and other immunosuppressant (on/off) to understand the combined effect of prednisone with other immunosuppressants. Patients receiving prednisone alone (n = 4) have highly dysregulated genes when compared to treatment-naive patients (n = 6), showing similar trends as seen in Figure 5A,B. Besides its anti-inflammatory properties, prednisone per se might trigger multiple signaling pathways, which may in part explain its detrimental effects when used long term (Figure 5C,D). Patients receiving prednisone along with other immunosuppressants (n = 9) have almost similar differential expression patterns as patients receiving only other immunosuppressants (n = 7), suggesting that other immunosuppressants on its own or in combination with prednisone might revert the dysregulation. Next, we compared patients’ nasal disease activity in addition to treatment status (Figure 6). Only one patient in the non-nasal disease group was off treatment and thus this group was excluded from this analysis. Here, it was observed that PR3+ AAV patients with inactive nasal disease reflected the general trend observed in PR3+ AAV patients, with inactive nasal disease patients on treatment (n = 5) having diminished differential expression levels when compared to those off treatment (n = 6) (Figure 6). On the contrary, patients with active nasal disease displayed little difference in expression levels between those on/off treatment (n = 5/n = 3).

By utilising differential gene expression analysis and prior knowledge of gene regulatory networks, we could estimate the activity of 271 transcription factors (TFs) in each PR3+ AAV patient—a process referred to as footprint analysis [13]. Footprint analysis revealed high absolute activity in the TFs BCL6, CEBPB, JUN SPI1 and SP1—all of which placed in top 15 TFs (Supplementary Table S2). Moreover, they were all present in the SA-PR3+ AAV interaction network. When looking at the average TF activity of these TFs in the on/off treatment groups they displayed a decrease in absolute activity in patients receiving immunosuppressant treatment when compared to those off treatment (Figure 4C). This trend was also seen in prednisone on/off treatment groups to a lesser extent with few exceptions (SMAD3, SP1 and ZNF639) (Figure 5C). The average TF activity was much higher in treatment-naive group, and prednisone treatment alone did not greatly reduce the activity of TFs, such as BCL6, JUN, SMAD3, SP1, SPI1, TBX21 and ZNF639 (Figure 5H). Other immunosuppressant treatment without prednisone has reduced overall TF activity and with prednisone the TF activity reduces even further with some exceptions (SMAD3, SP1 and ZNF639) (Figure 5I).

Patients with inactive nasal disease displayed a decrease in absolute TF activity when receiving when compared to those off treatment (Figure 6F). Overall, in active nasal disease the activity of these TFs showed little difference between those on/off treatment, but BCL6 and CEBPB showed increased absolute activity in those on treatment (Figure 6E).

## 3. Discussion

In this study, we created a network of genes whose dysregulation may be implicated in PR3+ AAV pathogenesis. This was done by combining prior knowledge of human–human and human–pathogen PPIs, gene regulatory networks, GWAS and drug–target interactions with publicly available transcriptomic data [10]. Captured in the network were a range of genes which have previously been reported to be dysregulated in PR3+ AAV. These included genes involved in the activation and priming of key immune effector cells (e.g., IL1B, IL2RA, IL6, IL10, IL32, TNF, MMP9, CD14 and SERPINA1), extracellular matrix remodelling and endothelial injury and repair (e.g., TIMP1, MMP9, S100A8/A9, ITGAM, ITGB2, ITGAX and ICAM1) highlighting the ability of our methodology to capture validated dysregulation in genes crucial to PR3+ AAV pathogenesis [11].

Unsupervised clustering of samples based on the expression of the PR3+ AAV disease network genes showed differences between healthy controls and diseased patients as well as between PR3+ AAV subgroups (Figure 2). Although clustering was unable to achieve complete separation of disease and healthy samples, the majority of PR3+ AAV patients that clustered most closely to healthy controls were those with inactive nasal disease receiving immunosuppressant treatment at the time of sampling.

Prior studies have suggested a possible role for SA in GPA pathogenesis and relapse [4,5,6]. To investigate this, we created a sub-network of the PR3+ AAV disease network identifying genes, biological processes and pathways (functional modules) which may interact with SA. As would be expected, genes associated with host-pathogen/-symbiont interactions and the immune response featured prominently in the SA-PR3+ AAV interaction network. Alongside these were genes associated with cell–cell/cell-matrix adhesion and the platelet response (Figure 3). Footprint analysis was used to estimate the activity of TFs in each patient highlighting the key regulators of these functional modules (Figure 4C). Proinflammatory cytokines such as such as TNF, IL1B and IL6 were present in the network and are a crucial part of innate immunity helping to promote inflammation in response to chemical and biological pathogenic stimuli (e.g., SA). They are also important in PR3+ AAV through their priming and activation of neutrophils and the resulting release of autoantigens [14]. The TFs CEBPB and SPI1 (also referred to as PU.1) were both shown to have increased activity in PR3+ AAV patients and have been reported to function together in promoting expression of IL1B (the most highly differentially expressed gene in our data) [15,16,17]. Interestingly, they have also both previously been reported to be upregulated in AAV patients [18]. Similarly, SP1 and JUN have been shown to function together to induce TNF expression in response to viral infection [19] and SPI1 has been shown to promote IL10 expression [20]—a cytokine often seen to be dysregulated in autoimmune diseases as in PR3+ AAV [21]. Furthermore, SPI1 is thought to regulate the expression of co-stimulatory proteins CD80 and CD86 in dendritic cells highlighting its role in regulating both innate and adaptive immune processes [22]. It has been reported that defective STAT5 activation and aberrant expression of BCL6 in naive CD4 T cells drives the skewed pathogenic CD4 effector immune response leading to increased production of cytokines IL21 and IL6 [23]. Also present in the network were a number of integrins and their interactors (e.g., ITGAM, ITGB1, ITGB2 and ICAM1) which play a role in mediating cell–cell and cell-matrix adhesion [24]. Many leukocytes utilise integrins to aid their migration to and activation at the site of inflammation [25]. Multiple studies have reported increased expression of key integrins such as those found in the network in monocytes and neutrophils derived from AAV patients or in response to ANCA exposure in vitro [26,27,28,29,30,31]. Indeed, the integrin receptor lymphocyte function-associated antigen 1 (LFA-1, partly encoded by ITGB2) has been suggested as a potential biomarker for AAV [30]. Moreover, these processes may be influenced by infectious agents such as SA, with evidence that LFA-1 is upregulated in response to lipopolysaccharide (LPS) recognition by the LPS-binding protein complex [32]—a complex involving CD14 and TLR4 both of which were upregulated in our data. The upregulation of integrins in the network is also reflected by the results of footprint analysis with increased activity of SPI1 which has been reported to promote myeloid-specific expression of integrins ITGAM (CD11b) and ITGB2 (CD18) [33,34]. Furthermore, over-representation of genes relating to platelet response may reflect the role platelets have in innate immunity and specifically their role in responding to microbial infection [35]. In response to the SA toxin α-haemolysin (also referred to as α-toxin), platelets were shown to release human β-defensin -1 (hBD-1)—an important antimicrobial peptide previously reported to be upregulated in GPA nasal mucosa [36]. Moreover, platelet-derived hBD-1 was shown to stimulate neutrophil extracellular trap formation, a process strongly implicated in PR3+ AAV [37].

The SA-PR3+ AAV interaction network revealed various ways through which SA may affect key disease processes in PR3+ AAV. The network incorporated 21 SA-human interactions involving 13 SA virulence factors including exoenzymes, exotoxins and SA surface proteins [12]. Nevertheless, without corresponding data on SA colonisation and expression of individual virulence factors the interactions shown in the network remain hypothetical. Previous reports have confirmed the presence of several virulence factors captured in the network (e.g., enterotoxins A and B (SEA/B encoded by *entA/B*) and toxic-shock-syndrome-toxin-1 (TSST-1 encoded by *tst*)) in SA isolates derived from GPA patients [6,38,39]. However, only the presence of TSST-1 has been associated with disease relapse [6].

Comparison of average differential expression levels of genes in the SA-PR3+ AAV network between patients on and off immunosuppressant treatment (excluding prednisolone) versus healthy controls revealed that patients on treatment showed reduced differential expression when compared to those off treatment (Figure 4A,B). Indeed, this trend was reflected in the average estimated activity levels of TFs, with lower absolute activity levels in patients on treatment when compared to those off treatment (Figure 4C). Comparison of SA-PR3+ AAV interaction networks between patients with or without prednisone and other immunosuppressants revealed that patients receiving other immunosuppressant treatment either mono or in combination with prednisone have reduced differential expression when compared to patients not treated with other immunosuppressants (Figure 5C–F). This trend was also reflected in the average estimated activity levels of TFs, with lower absolute activity levels in patients receiving other immunosuppressants when compared to those off other immunosuppressant treatment (Figure 5H,I). This is consistent with the results of clustering analysis, which placed disease patients receiving immunosuppressive treatment closest to healthy controls when looking at the expression of the larger PR3+ AAV network. The network being dysregulated despite prednisone treatment may suggest that besides its beneficial effects prednisone might trigger multiple signaling pathways, ultimately causing tissue damage and other detrimental effects, such as development of cardiovascular disease.

Further stratification of patients according to their nasal disease activity at the time of sampling showed that patients with inactive nasal disease reflected the general trend observed for PR3+ AAV, however, this trend was not observed in those with active nasal disease with treatment showing no obvious reduction in differential expression or absolute TF activity (Figure 6). The maintained dysregulation seen in active nasal disease patients despite receiving immunosuppressant treatment may be influenced by a range of factors including SA colonisation with endonasal activity having previously been associated with SA colonisation in GPA patients [4]. However, without corresponding parallel data on SA colonisation this hypothesis was unable to be tested. Moreover, in a study investigating the composition of the nasal microbiome of GPA patients and healthy controls there was no significant difference between healthy controls and GPA patients receiving non-glucocorticoid immunosuppressants whereas a significant difference (i.e., dysbiosis) was observed in GPA patients not receiving such treatment [40]. This suggests that non-glucocorticoid immunosuppression may affect not only the patient’s transcriptome as observed in our data but also the nasal microbiome—both having the potential to modulate PR3+ AAV disease processes.

Overall, this study provides insight that nasal disease is characterised by the upregulation of key inflammatory pathways, also known to be induced in blood samples. Upregulated genes comprise a set of genes involved in extracellular matrix remodeling, leukocyte adhesion perpetuating the inflammatory response and endothelial injury, key factors relevant to induce chronic damage as seen in PR3-ANCA vasculitis with nasal disease. Mucosal damage might be triggered by environmental factors and further studies looking into the role of SA in this context need to be undertaken. Our analysis shows that in nasal disease several SA virulence factors are represented, likely causing a breakdown of the nasal mucosal barrier, and of relevance to induce active disease. Further studies will need to focus on the impact of specific local and systemic therapies, ranging from mupirocin ointment to effects exerted by novel therapies, such as the C5aR1 inhibitor avacopan.

To conclude, our network-based methodology was able to capture validated dysregulation in genes important to PR3+ AAV pathogenesis that may help to prioritise diagnostic markers and therapeutic candidate genes [41,42]. We highlighted how these key genes and their associated processes may be affected by interactions with SA. Lastly, our results showed immunosuppressant treatment reduced dysregulation in genes important in PR3+ AAV and potential SA interactions in inactive nasal disease patients as evidenced by differential expression, footprint and clustering analysis but not in patients with active nasal disease. However, parallel data on SA colonisation and the expression of the relevant virulence factors would be needed to derive more robust mechanistic insights.

## 4. Materials and Methods

### 4.1. Disease Network Construction

#### Data Acquisition and Pre-Processing

Data acquisition and pre-processing were conducted in Rstudio (version 4.0.2). Transcriptomic data in the form of microarray intensities were obtained from the Gene Expression Omnibus database [43] (GEO accession number GSE119136 [10]). The raw data was normalised using Robust Multichip Average and Log2 transformed via the affy R package (version 1.66.0). Probe summarisation was carried out according to Affymetrix Chip Definition File Human Gene 1.0 ST v1.r4.

### 4.2. Microarray Data Analysis

From the pre-processed data, we selected PR3+ ANCA patients (as defined in the original study [10]) and healthy controls. Differential gene expression analysis comparing the PR3+ ANCA patients versus healthy controls was performed using mixed linear regression models via the limma R package (version 3.44.3) [44] adjusting for immunosuppressant treatment status and batch effect. Treatment status was defined as “on treatment” versus “off treatment” based upon use of immunosuppressants (excluding prednisolone) as defined in ‘immune_or_nasal’ column in the meta data of the original study [10]).

### 4.3. Predicting TF Activity (Footprint Analysis)

Transcription factor (TF) activity estimation was performed using the viper R package (version 1.24.0) [45] with limma moderated t-scores used as gene expression signatures. TF-target interactions were obtained from the DoRothEA database [46] using the DoRothEA R package (version 1.3.0). Interactions of A, B and C confidence levels were kept. The eset.filter parameter was set to FALSE and only TFs with at least 5 measured transcripts were included.

### 4.4. Finding Genes of Interest

Three lists of genes/proteins potentially of importance in GPA—and specifically in PR3+ AAV- were compiled. List 1 was derived from Table S7 of KS Lee, A Kronbichler, DF Pereira Vasconcelos, FR Pereira da Silva, Y Ko, YS Oh, M Eisenhut, PA Merkel, D Jayne, CI Amos, et al. [47], which contains statistically significant GWAS genes associated with PR3+ AAV, GPA and AAV. List 2 contained all human proteins listed in the Host Pathogen Interaction Database (HPIDB) [12] as known interactors with SA proteins. List 3 contained drugs used clinically or investigationally to treat GPA and their corresponding targets obtained through OpenTargets [48] and manual literature search.

### 4.5. Constructing a Reference PPI Network

To create the PR3+ AAV disease network, a reference PPI was first constructed onto which the selected genes could subsequently be mapped. The reference network was constructed by integrating directed PPIs from two sources: Diffuse2Direct consensus network published in association with D Silverbush and R Sharan [49], OmniPath PPI DB [50] (accessed via OmnipathR (version 1.2.1)) filtered to include only directed and high confidence interactions (defined as consensus_direction = 1 and nsources >3). Network analysis and construction were done in Python (version 3.8.3) using the NetworkX package (version 2.4).

### 4.6. Selecting Disease Genes

To select the genes included in the PR3+ AAV disease network, moderated t-scores for each gene were mapped onto their corresponding proteins in the reference PPI. Proteins without expression values were removed from the network. Once mapped, each gene’s expression value was adjusted taking into account the expression of its neighbours in the network (as described in H Han, S Lee and I Lee [51]). The top 300 genes (according to their network adjusted value) whose expression is regulated by any of the top 50 TFs (according to their absolute estimated activity score), the top 50 TFs, genes that had a network adjusted expression value in the top 150 but were not regulated by any of the top 50 TFs and all drug targets present in the reference PPI were selected. Additionally, SA interacting proteins and GWAS genes (lists 1 and 2, respectively) were selected if they were a direct neighbour to any of the previously selected genes. All selected genes and the interactions between them formed the initial disease network.

### 4.7. Augmenting Disease Network

The initial disease network was then augmented using the DIseAse MOdule Detection (DIAMOnD) algorithm run for 50 iterations using the selected proteins as ‘seed genes’ and the reference PPI as the network [52]. To aid network connectivity further ‘connector’ genes were added. This was done by finding unconnected genes (defined as being unconnected from the largest connected component of the network) and iterating over each unconnected gene’s list of neighbours. Any neighbours which themselves had a neighbour in the largest connected component were added completing the disease network.

### 4.8. Disease Network Analysis

#### Gene Set Enrichment Analysis Using g:Profiler

Gene set enrichment analysis (GSEA) was carried out for the disease network using g:profiler via the online web-tool [53]. Inputted into g:profiler were the genes making up the disease network. The query was run as an ordered query with input genes ranked according to their network adjusted expression value. Gene Ontology biological processes (GO:BP) and Reactome biological pathways were used as the annotated gene sets. All other settings were kept as default. The results of g:profiler were filtered removing terms with a term size <5 and >350 and a g:SCS >0.05. The size filtered ‘.gem’ file was downloaded and subsequently edited in R studio to remove terms that did not contain any SA interacting genes.

### 4.9. GSEA Visualisation, Disease Network Filtering and SA-PR3+ AAV Interaction Network Creation

GSEA visualisations were done in Cytoscape [53]. After filtering, the ‘.gem’ was inputted into EnrichmentMap (version 3.3.0) setting the analysis type to ‘Generic/Enrichr/gProfiler’ creating a network of GO:BPs and Reactome pathways as nodes and edges representing shared genes. To find the most relevant terms to SA involvement in PR3+ AAV, we selected a subset of terms which had the highest number of SA interacting genes associated with them. This was done iteratively. Terms were first arranged in descending order according to the number of SA genes associated with them. Starting at the term with the highest number of SA interacting genes, each term’s genes were read and genes which were also present in the disease network were added to a list. When the number of top 50 TFs in this list exceeded or equalled 10, no further terms were added. The resulting list of genes were mapped to the reference PPI creating a sub-network of the disease network referred to as the SA-PR3+ AAV interaction network. To find the processes more widely associated with the selected terms (and their genes), we found the term’s neighbours in the GSEA network and created a sub-network from this. The sub-network of terms was then clustered and labelled using the AutoAnnotate app (version 1.3.4) in Cytoscape.

### 4.10. Patient Comparisons in SA-PR3+ AAV Interaction Networks

Differential gene expression analysis was carried out for each PR3+ AAV patient vs. healthy controls taking the log2 transformed fold-change (log2FC) in the normalised intensities for each gene. Subsequently, footprint analysis was carried out for each PR3+ AAV patient as previously described instead using the log2FC as the gene expression signature (as opposed to moderated t-scores). These values were mapped to the relevant genes/TF in Cytoscape allowing colour coding and size scaling of the network.

## Figures and Tables

**Table 1:** Baseline characteristics and treatment of patients with proteinase 3 (PR3)-ANCA vasculitis, subdivided in those with active and inactive nasal disease activity, and those with no nasal involvement.

	Active Nasal Disease (8 Patients)	Inactive Nasal Disease (11 Patients)	No Nasal Involvement (7 Patients)
Mean age (years)	41.5	48	60.3
Disease duration (years)	2.3	5.6	7
BVAS (WG; mean)	5.3	0	0
VDI	1.3	1.5	1
Disease characteristic (limited/severe) (n; %)	5 limited (62.5) 3 severe (37.5)	4 limited (36.4) 7 severe (63.6)	1 limited (14.3) 6 severe (85.7)
Relapsing disease (n; %)	3; 37.5 (range 1–6)	6; 54.5 (range 1–7)	3; 42.9 (range 1–2)
Female sex (%)	62.5	18.2	57.1
Smoking status	Never: 6 Former: 2	Never: 8 Former: 2 Current: 1	Never: 1 Former: 5 Current: 1
On prednisone (n; %)	7; 87.5	3; 27.3	3; 42.9
Mean prednisone dose, mg	24.9	1.3	2.9
Number taking other immunosuppressants (n; %)	5; 62.5	5; 45.5	6; 85.7
Rituximab use (n; %)	1; 12.5	0; 0	2; 28.6

## Data Availability

The publicly available transcriptomics datasets analysed in this study are available at Gene Expression Omnibus accession number GSE119136 (https://www.ncbi.nlm.nih.gov/geo/). The scripts used in this analysis are available on reasonable request.

## Supplementary Materials

The following supporting information can be downloaded at: https://www.mdpi.com/article/10.3390/ijms24031822/s1, Supplementary Table S1—Table containing differential expression and network statistics for genes in the disease network outputted by our NBA pipeline; Supplementary Table S2—Table containing all TFs and their estimated activity score generated in our footprint analysis outputted from VIPER in R studio.
